# A Novel Laser Refractive Surgical Treatment for Presbyopia: Optics-Based Customization for Improved Clinical Outcome

**DOI:** 10.3390/s17061367

**Published:** 2017-06-13

**Authors:** Bojan Pajic, Brigitte Pajic-Eggspuehler, Joerg Mueller, Zeljka Cvejic, Harald Studer

**Affiliations:** 1 Eye Clinic Orasis, Swiss Eye Research Foundation, CH-5734 Reinach, Switzerland; bpajic@datacomm.ch (B.P.); brigitte.pajic@orasis.ch (B.P.-E.); joerg.mueller@nova-optik.ch (J.M.); 2 Department of Physics, Faculty of Sciences, University of Novi Sad, Trg Dositeja Obradovica 4, 21000 Novi Sad, Serbia; zeljkac@uns.ac.rs; 3 Division of Ophthalmology, Department of Clinical Neurosciences, Geneva University Hospitals, CH-1205 Geneva, Switzerland; 4 Faculty of Medicine of the Military Medical academy, University of Defence, 11000 Belgrade, Serbia; 5 OCTlab, Department of Ophthalmology, University of Basel, CH-4001 Basel, Switzerland

**Keywords:** presbyopia, LASIK, presbyLASIK, uncorrected visual acuity

## Abstract

Laser Assisted in Situ Keratomileusis (LASIK) is a proven treatment method for corneal refractive surgery. Surgically induced higher order optical aberrations were a major reason why the method was only rarely used to treat presbyopia, an age-related near-vision loss. In this study, a novel customization algorithm for designing multifocal ablation patterns, thereby minimizing induced optical aberrations, was used to treat 36 presbyopic subjects. Results showed that most candidates went from poor visual acuity to uncorrected 20/20 vision or better for near (78%), intermediate (92%), and for distance (86%) vision, six months after surgery. All subjects were at 20/25 or better for distance and intermediate vision, and a majority (94%) were also better for near vision. Even though further studies are necessary, our results suggest that the employed methodology is a safe, reliable, and predictable refractive surgical treatment for presbyopia.

## 1. Introduction

Laser Assisted in Situ Keratomileusis (LASIK) is proven to be a safe, fast, and reliable procedure for corneal refractive surgery. The procedure comprises three steps: (i) cutting a thin flap on the outer corneal surface, (ii) ablating tissue underneath the flap with an excimer laser, and (iii) putting the flap back into place on the stromal bed. Only a relatively small number of side effects, such as dry-eye, halos, corneal ectasia, and epithelial ingrowth under the flap, have been reported. Modern excimer laser systems have demonstrated their ability and performance in treating various ametropic conditions, such as nearsightedness (myopia), farsightedness (hyperopia), and astigmatism. By 2009, more than 27 million eyes had successfully been treated with LASIK refractive surgery all around the world.

Even though various approaches—based on diverse technologies and methodologies—for the treatment of age-related far or nearsightedness have been proposed and documented in literature, effective presbyopia treatment remains a challenge in modern eye care. State-of-the-art treatment involves the implantation of multifocal intraocular lenses [1,2], which by nature is a highly invasive surgical procedure, and postoperative refraction planning remains highly complex. Other approaches such as mono-vision corneal procedures [3,4] use excimer lasers and LASIK to treat presbyopia, but these methods are unfortunately not well tolerated by many subjects. Corneal inlay implantation [5,6], intrastromal femtosecond laser corrections [7], or other excimer laser-based presbyopia treatments [8] have been used in clinics with some success over the last few years.

In any such procedure, besides restoring the patients’ vision and ability to see far- as well as near-distance objects, the focus should always be that (i) the treatment is reversible and (ii) that postsurgical enhancement, or touch-up, is always possible. Generally speaking, corneal approaches are the least invasive, and are highly accurate and safe procedures. Furthermore, they omit risks that are inherent to the intraocular procedure. It has previously been reported that multifocal LASIK treatments, sometimes also called presbyLASIK treatments, in the cornea showed good refractive results for near, intermediate, and distance vision in hyperopic presbyopia patients [8,9,10]. The good results of those studies suggest that postoperative refraction was easily predictable, and that presbyLASIK was well tolerated by the study subjects. However, because LASIK ablation patterns for myopic eyes are fundamentally different from those of hyperopic treatments, this prospective study aimed to assess the performance of presbyLASIK customized multifocal procedures for myopic presbyopia patients.

## 2. Materials and Methods 

This prospective, single-surgeon study of myopic presbyopia treatments with a multifocal presbyLASIK procedure included 72 eyes of 36 patients. All eyes underwent pre- and postoperative full clinical biomicroscopical examination, Orbscan IIz corneal topography (BAUSCH + LOMB, TECHNOLAS Perfect Vision GmbH, Munich, Germany) and Zywave II wavefront aberrometry analysis (BAUSCH + LOMB, Rochester, NY, USA). Additionally, monocular and binocular uncorrected near (UNVA), intermediate (UIVA), and distance (UDVA) visual acuity were assessed using a LogMAR chart. For all examinations, the eyes had undilated pupils, and were conducted preoperatively, as well as one week, one month, three months, and six months postoperatively.

**Figure 1:**
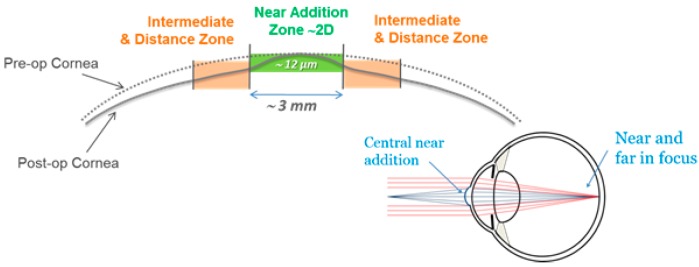
Schematic description of the Supracor treatment. The multifocal ablation pattern combines a regular ablation for the subjects’ ametropic condition (shown in orange) with a 3-mm central zone near the vision add-on (shown in green). The add-on typically adds 2 diopters of refractive power to the central cornea.

All study subjects underwent Supracor refractive surgery, operated with a 217P Excimer laser (BAUSCH + LOMB, TECHNOLAS Perfect Vision GmbH, Munich, Germany). The dominant eye of each individual subject was targeted plano for far vision (0.0 diopters), while the respective non-dominant eye of the same subject was targeted at −0.5 diopters. All LASIK flaps were created with a Ziemer LDV femtosecond laser platform (Ziemer Ophthalmology, Port, Switzerland), and had a superior hinge and a thickness of 110 microns. All treatments were planned and executed in two main steps: first, the normal ablation pattern for the myopic condition of the subject’s eyes were applied to the cornea, according to the surgeon’s nomogram, and by targeting the mean refractive spherical equivalent (MRSE) to be optimal for distance vision. Second, the aforementioned 3-mm zone near the addition was applied (see Figure 1) to create the extra refractive power in the central cornea. The resulting multifocal shape allowed the patient to have clear vision over a wide range of depth of focus.

**Figure 2:**
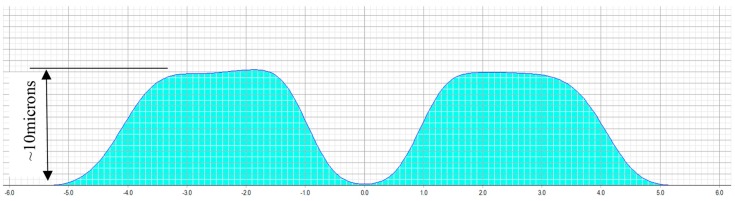
Typical multifocal ablation profile of a presbyopia-only treatment. The schematic indicates the amount of ablated tissue (blue area), with respect to the distance from the center of the cornea. Such a profile creates a steep central zone of 3 mm, providing extra refractive power for near-vision.

**Figure 3:**
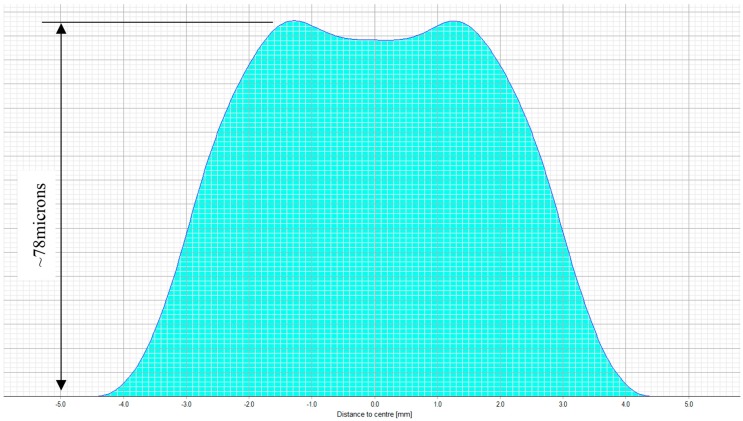
Typical multifocal ablation profile of a combined myopic and presbyopic treatment. The schematic indicates the amount of ablated tissue (blue area), with respect to the distance from the center of the cornea. Such a profile corrects the subjects’ nearsightedness, as well as creates a steep central zone of 3 mm, providing extra refractive power for near-vision.

The treatment planning software of a 217P laser system calculates a multifocal ablation pattern by combining the normal distance vision treatment plan for the ametropic condition of the subject, with a near vision addition in the 3-mm central zone of the treatment (see Figure 2 and Figure 3). On one hand, the normal distance vision treatment thereby uses an adaptation of the original Munnerlyn formula [11] to flatten the overall cornea in the peripheral and paracentral zone. This flattening is customized to the individual eye, to reduce the cornea’s refractive power and to bring the focus point of the eyes optical system right onto the retina. The near-vision addition, on the other hand, creates a small central region of higher curvature and hence a higher refractive power. The higher power is customized such that close-by targets are well focused on the retina. The hypothesis of multifocal ablations is that the brain is capable of blending the retinal images, and hence enables the subject to have good near, intermediate, and distance visual acuity. In order to prevent undesired optical side effects from the transition between the small area of high curvature and the flatter corneal region, a proprietary customization algorithm ensures that postoperative spherical aberrations are avoided.

## 3. Results

**Figure 4:**
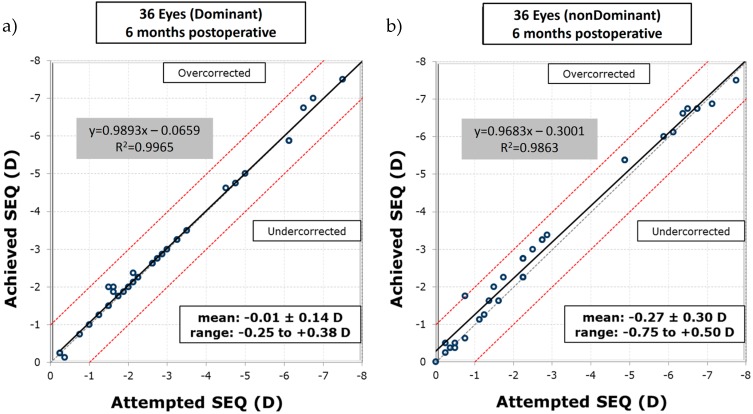
Attempted versus achieved spherical equivalent (SEQ) of (**a**) dominant and (**b**) non-dominant eyes, six months postoperatively. The regression lines in each graph indicate a small undercorrection for low values and a slight overcorrection for high values of spherical equivalent. SEQ: spherical equivalent; D: unit of refractive power, diopters.

All 72 surgical procedures were successfully executed, no side effects were detected, and not a single complication occurred during the study period. Measured on the LogMAR scale with respect to preoperative visual acuity (near: 0.29 ± 0.3, intermediate: 0.38 ± 0.36, distance: 0.75 ± 0.45), the visual acuity of subjects six months after surgery improved significantly, for near (−0.01 ± 0.11, *p* = 5 × 10^−6^), intermediate (−0.01 ± 0.07, *p* = 2 × 10^−7^), and distance vision (−0.09 ± 0.09, *p* = 4 × 10^−12^). Furthermore, the visual acuity of all eyes remained stable postoperatively after six months. The excimer laser system applied the planned ablation patterns at a very high precision, as the differences between the targeted and achieved spherical equivalent (SEQ), given in the unit of refractive power—diopters (D), at six months postoperative were −0.01 ± 0.14 D (standard deviation—SD) with an r-square of $R^{2}=0.997$ and −0.27 ± 0.30 D (SD) with $R^{2}=0.986$, for dominant and non-dominant eyes, respectively (see Figure 4). Moreover, 72% of the dominant eyes had an SEQ accuracy to the target of ±0.13 D, 17% were between +0.14 D and +0.50 D, and 11% were between −0.14 D and −0.50 D. In the non-dominant eyes, 42% were within ±0.13 D, 47% were within +0.14 D and +0.50 D, 8% were within −0.14 D and −0.50 D, and 3% were between +0.51 D and 1.00 D. 

**Figure 5:**
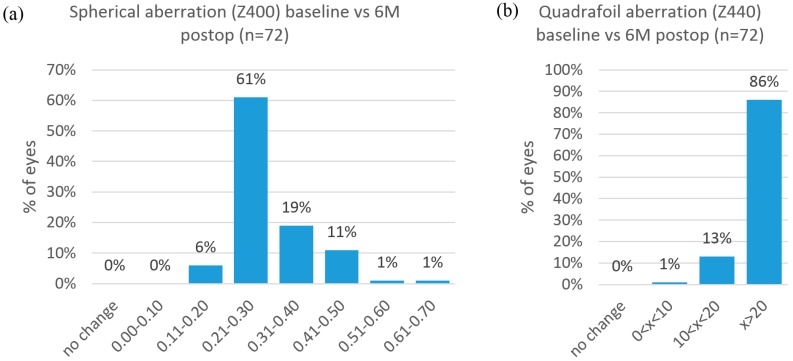
Surgically induced spherical (**a**) and quadrafoil (**b**) aberrations in micrometers, compared preoperatively (baseline) to postoperatively (postop). The treatment induced 0.21 to 0.40 microns of spherical aberration (Z400) and more than 20 microns of quadrafoil aberration (Z440) in most eyes.

The surgical treatment reduced spherical, and quadrafoil aberration in most eyes. Fifty-eight out of the 72 eyes had a decreased spherical aberration between 0.21 to 0.40 microns. Nine eyes had a decrease of quadrafoil aberration between 10 and 20 microns, and 62 eyes had a decrease of more than 20 microns of quadrafoil aberration (see Figure 5).

**Figure 6:**
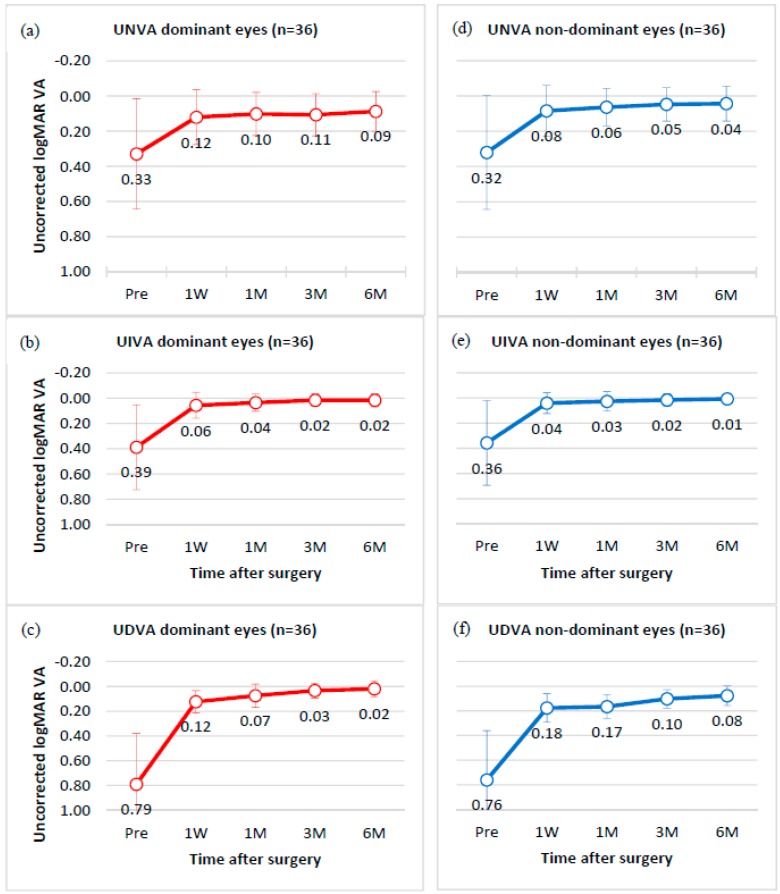
Mean and standard deviation of monocular uncorrected visual acuity for near (UNVA), intermediate (UIVA), and distance vision (UDVA) for 36 dominant (**a**–**c**) (shown in red), and 36 non-dominant (**d**–**f**) (shown in blue) eyes. Values are given preoperatively, and at one week (1 W), one month (1 M), three months (3 M), and six months (6 M) postsurgical follow-up.

**Figure 7:**
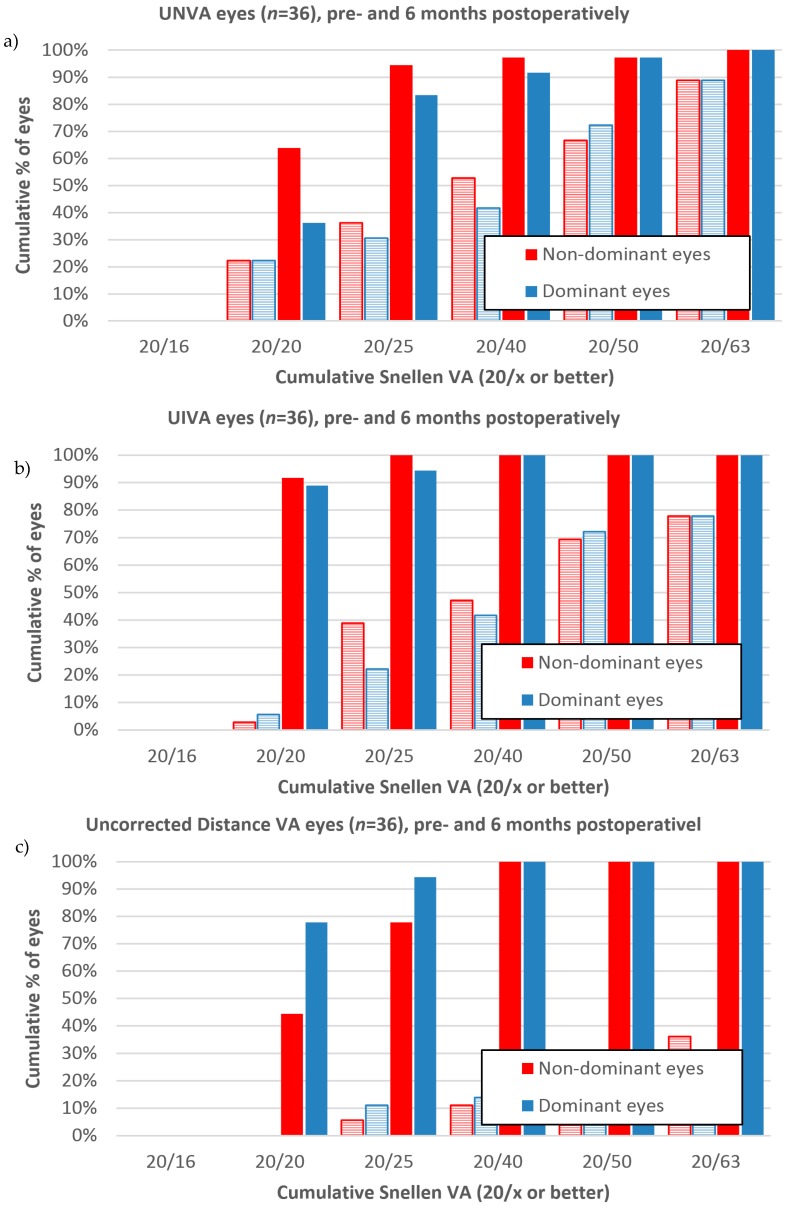
Cumulative percentage of subjects with 20/x dominant and non-dominant near (**a**), intermediate (**b**), and distance (**c**) visual acuity for 36 subjects. Dashed bars are preoperative, and solid bars are six-months postoperative data. Each percentage value is to be interpreted as 20/x vision or better.

**Figure 8:**
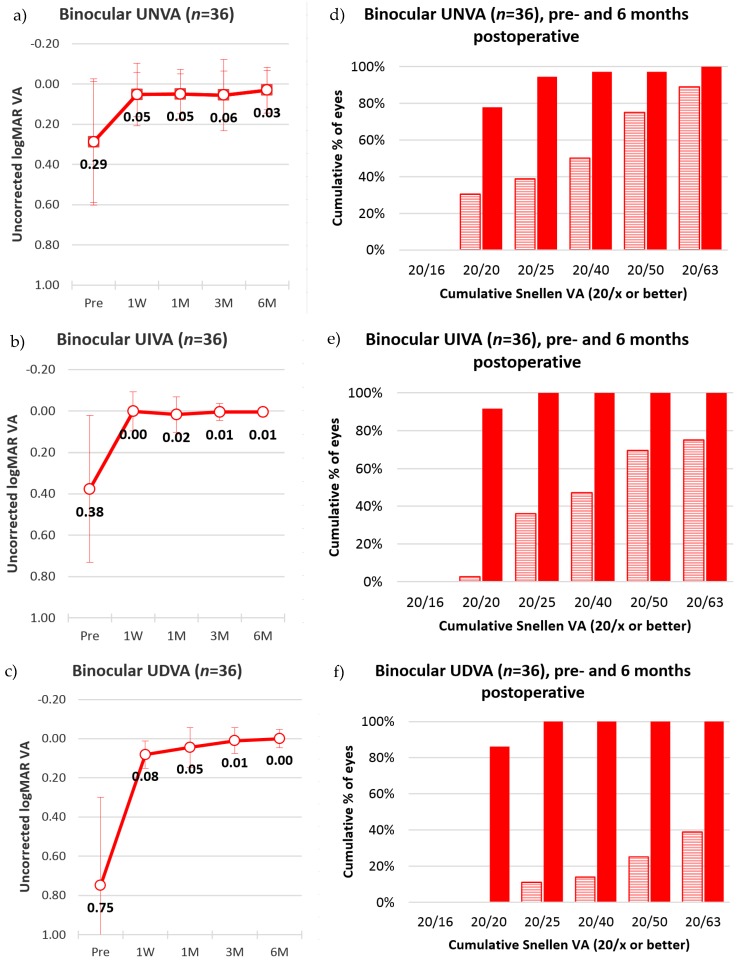
(**a**–**c**) Mean and standard deviation of binocular uncorrected visual acuity UNVA, UIVA, and UDVA vision for 36 subjects. Values are given preoperatively, and at one week (1 W), one month (1 M), three months (3 M), and six months (6 M) postsurgical follow-up. (**d**–**f**) Cumulative percentage of subjects with 20/x binocular near (UDVA), intermediate (UIVA), and distance (UDVA) vision for 36 subjects. Each percentage value is to be interpreted as 20/x vision or better.

Figure 6 presents the monocular mean and standard deviation of visual acuity before and after the surgery, for near, intermediate, and distance vision of all 36 subjects, in the LogMAR scale. At the six-month post-surgical follow-up for the dominant eyes, uncorrected near, intermediate, and distance visual acuity of 0.09 ± 0.11 (SD), 0.02 ± 0.04 (SD), and 0.02 ± 0.07 (SD) were observed, respectively. Meanwhile, for the non-dominant eyes, the six-month follow-up showed 0.04 ± 0.10 (SD), 0.01 ± 0.03 (SD), and 0.08 ± 0.08 (SD), for near, intermediate, and distance uncorrected visual acuity. Figure 7 indicates that while 36% of the dominant eyes had 20/20 uncorrected near visual acuity or better, six months after the intervention, 64% of the non-dominant were at 20/20 for uncorrected near visual acuity. The results in the figure further show that all eyes had 20/40 uncorrected visual acuity or better for distance and intermediate vision, while only 92% of the dominant and 97% of the non-dominant eyes had 20/40 uncorrected visual acuity for near vision. Figure 8 shows that the mean binocular visual acuity was at 0.03 ± 0.1 (SD), 0.01 ± 0.02 (SD), and 0.00 ± 0.05 (SD) for uncorrected near, intermediate, and distance vision, six months after the treatment. Further, the results show that 78% of the subjects had 20/20 uncorrected near visual acuity, 92% had 20/20 uncorrected intermediate visual acuity, and 86% had 20/20 distance visual acuity, at six months after surgery. 

## 4. Discussion

A prospective, single-center, single-surgeon clinical study on multifocal, myopic, presbyLASIK treatments, with an excimer laser, was carried out on 36 subjects. The applied treatment targeted the dominant eye to plano and the non-dominant eye to −0.5 diopters, while introducing a near addition of two diopters in the central cornea to increase the depth of focus. 

Results suggest that the applied presbyLASIK procedure is an effective treatment for presbyopia. All study subjects had an uncorrected visual acuity of 20/40 or better for near, intermediate, and distance vision. Over 90% of the subjects were at 20/25 (LogMAR ≤ 0.10) or better, and about 80% were even at 20/20 (LogMAR ≤ 0.00) or better, again for the whole range of near, intermediate, and distance vision. In contrast to that, multifocal lens implantation results in the literature showed LogMAR 0.09 ± 0.08, LogMAR 0.10 ± 0.11, and LogMAR 0.07 ± 0.05, for near, intermediate, and distance vision [2], respectively, and in some cases even required spectacle correction for acceptable distance vision [1]. Mono vision treatments only showed 20/20 or better in 36.7% of the subjects for near, and in 31.1% of the subjects for distance visual acuity [3]. Results of intracorneal inlays were comparable for uncorrected near vision [6], and distance vision was not reported.

As expected, while the dominant eyes in this study generally had higher monocular visual acuity for distance vision, the non-dominant eyes performed better in monocular near vision. Presumably, the brains of the study candidates were capable of blending the two distinct monocular images into a binocular image, electively focusing on near, intermediate, and distant targets. This circumstance is supported by the thoroughly high satisfaction of all of the study patients. 

Generally speaking, the excimer procedure employed in this study was less invasive compared to other presbyopia methods, such as refractive lens implantation. LASIK is a very well accepted and extensively proven procedure. It features high precision in positioning of the correction, in refractive outcome, as well as in predictability of the result. A key factor for this is the high precision and repeatability of flap quality and thickness [12]. A remaining problem with LASIK-based presbyopia treatments, however, is that the multifocal ablation may induce unwanted optical aberrations. Multifocal ablations usually are composed of a correction for distance vision (myopic or hyperopic), and a near addition. Lower order optical aberrations, specifically spherical aberrations, may be caused by the distance correction ablation. Additional higher order aberrations stemming from the transition between the distance treatment zone and the near addition might be induced, even though they may be outside of the region of the central addition, yet inside the optical zone of the distance vision correction. It seems apparent that the resulting refractive surface might evoke unfavourable optical aberrations [9,13,14]. Our wavefront results suggest that the customization algorithm for aberration reduction works very well. In almost all cases, spherical (Z400) as well as quadrafoil (Z440) aberrations were significantly reduced by the treatment.

The thoroughly positive results with the LASIK-based presbyopia treatment in this study can, at least partially, be attributed to the aspherical customization algorithm. The algorithm utilized the K-readings as well as the conic constant (Q) of the cornea to minimize the induction of adverse optical aberration effects [8,10,12]. In addition, using LASIK provides the option to re-touch the treatment with relative ease, and therefore has the potential to remove or enhance the presbyopic addition [9]. Even though further studies with more surgical cases are necessary to confirm these results, the presbyLASIK treatment employed in this study has great potential to become a gold standard for the treatment of presbyopia, as it safe and shows reliable, predictable, and satisfying outcomes.
